# Efficacy of the "critical view/open book" concept for implementing standardized right hemicolectomy with complete mesocolic excision—results from a series of minimally invasive surgical training courses

**DOI:** 10.1007/s00464-026-12901-7

**Published:** 2026-05-29

**Authors:** Marvin Heimke, Tillmann Heinze, Heiko Aselmann, Matthias Biebl, Maximilian Brunner, Jonas Johannink, Werner Kneist, Benedikt Reichert, Julius Pochhammer, Christoph W. Strey, Andreas Türler, Christoph Wullstein, Stefan Benz, Thilo Wedel

**Affiliations:** 1 Institute of Anatomy, Center of Clinical Anatomy, Kiel University, Kiel, Germany; 2 Department of General and Visceral Surgery, Diakovere Henriettenstift, Hannover, Germany; 3 Department of General, Visceral, Thoracic and Transplantation Surgery, Ordensklinikum Linz, Linz, Austria; 4 Department of Surgery, University Hospital Erlangen, Erlangen, Germany; 5 Department of General, Visceral and Transplantation Surgery, University Hospital Tübingen, Tübingen, Germany; 6 Department of General, Visceral and Thoracic Surgery, Hospital Darmstadt, Darmstadt, Germany; 7 Department of General, Visceral, Thoracic, Transplantation and Pediatric Surgery, University Hospital Schleswig-Holstein Campus Kiel, Kiel, Germany; 8 Department of General and Visceral Surgery, Hospital Clementinenhaus Hannover, Hannover, Germany; 9 Department of General and Visceral Surgery, Johanniter Hospital Bonn, Bonn, Germany; 10 Department of Visceral and Minimal Invasive Surgery, Helios Hospital Krefeld, Krefeld, Germany; 11 Kurt-Semm Center for Laparoscopic and Robotic Surgery, University Hospital Schleswig-Holstein Campus Kiel, Kiel, Germany

**Keywords:** Complete mesocolic excision, Critical view concept, Minimally invasive surgery, Open book model, Right hemicolectomy, Standardization, Surgical training course

## Abstract

**Background:**

Since the introduction of right hemicolectomy with complete mesocolic excision (CME) the implementation of minimally invasive CME as standard of care still remains challenging. Among several established guidelines for minimally invasive CME, the “critical view/open book” concept has been developed to provide a stepwise standardization with safety checkpoints for teaching purposes. The present study evaluates this concept during its practical application in a large series of surgical training courses.

**Methods:**

In this prospective observational study, seven consecutive 2-day surgical training courses based on the “critical view/open book" concept were offered to 125 attendees. Lectures on the rationale, surgical anatomy, techniques, and pitfalls of minimally invasive CME were followed by demonstration of predissected anatomical specimens. Tutored laparoscopic and robotic right hemicolectomies with CME were performed on body donors strictly adhering to the "critical view/open book" concept with subsequent quality assessment of resected specimens. A post-training questionnaire evaluated the "critical view/open book" concept related to its applicability, usefulness, efficacy, and adoption.

**Results:**

The "critical view/open book" concept could be fully/mostly applied by 85.9% of the attendees during the surgical training courses. The most valuable insights gained were related to the retrocolic fascial system, vascular variations including the gastropancreaticocolic trunk, and the extent of mesocolic excision. Although most attendees had no previous experience with the "critical view/open book" concept, 83.3% of resected specimens fulfilled the highest quality standard (type 0, Benz classification). Intention to adopt the "critical view/open book" concept into clinical practice was stated by 80.9% of those attendees not previously familiar with this concept.

**Conclusion:**

The "critical view/open book" concept applied in surgical training courses proved to be an efficient teaching tool for minimally invasive CME and, thus, may be integrated into training curricula. Follow-up studies are needed to confirm its impact on long-term adoption and clinical outcome.

**Graphical abstract:**

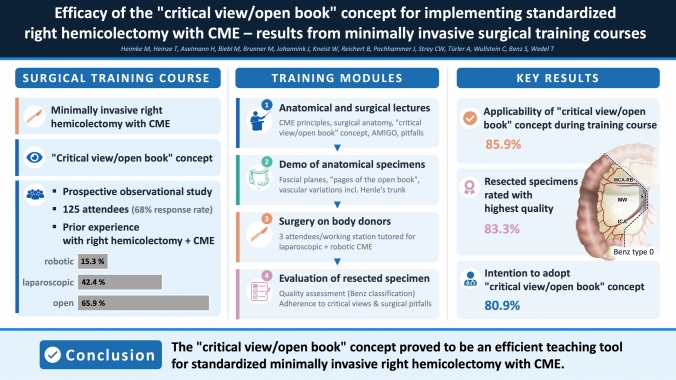

**Supplementary Information:**

The online version contains supplementary material available at 10.1007/s00464-026-12901-7.

Since the seminal paper by Hohenberger et al. describing the open technique for right hemicolectomy with complete mesocolic excision (CME) for colon cancer [1], the principles of CME (intact mesocolon, central vascular ligation, D3-lymphadenectomy) have been widely adopted among the surgical community. Although right hemicolectomy with CME remains a challenging procedure due to its comparably high complication risks [2], the feasibility of minimally invasive surgery has been confirmed for both laparoscopic and robotic approaches with similar oncological outcomes compared to open surgery [3].

Nevertheless, surgical approaches (e.g., medial, subileal, uncinate-first, supracolic, vessel-first approach) and procedural steps vary widely depending on institutional/regional preferences and they influence clinical and oncological outcomes [4, 5]. The lack of standardization prompted an international expert group to establish a Delphi-based consensus on a structured description of the surgical technique and the training framework essential for CME. While no consensus was reached for a favored surgical approach/technique, all experts highlighted "the real need for a structured training pathway that encompasses assessment of performance to promote quality standards" [6]. In line with these recommendations, a national multicentre prospective interventional sequential cohort study was set up in the Netherlands to determine a standardized surgical technique for right-sided colon cancer, provide hands-on surgical training courses, and implement the procedure in selected patient cohorts [7].

Accordingly, a German working group of surgeons experienced in minimally invasive surgery established a consensus-based standardized surgical technique for right-sided hemicolectomy with CME by providing a "critical view/open book" concept [8]. This concept comprises 8 critical views of safety allowing for a clear identification of surgical dissection planes to mobilize the colon/mesocolon and an optimized control during central vascular ligation and lymphadenectomy.

The working group has applied the "critical view/open book" concept in a nationwide surgical training course offered twice a year by the German Society for General and Visceral Surgery (DGAV) in order to promote a safe adoption and wider implementation of minimally invasive CME. The surgical training course consisted of theoretical and practical modules including a tutored training of both laparoscopic and robotic surgical interventions on body donors. The didactic concept adhered closely to the consensus statements on technical steps and training implications for CME [6] and to the protocol (phase 3: "training") provided by the national multicentre prospective interventional sequential cohort study for the implementation of an optimal standardized technique for right‑sided colon cancer [7].

This study was aimed at evaluating whether this surgical training course enabled (1) an understanding of the general rationale and principles of CME, (2) the clarification of the topographic anatomy and landmarks relevant for CME, (3) the practical applicability of the "critical view/open book" concept in a body donor setting, (4) the resection of high-quality CME specimens, (5) the adoption and implementation of the "critical view/open book" concept into surgical practice as the standard of care.

## Materials and methods

The prospective observational educational study was conducted at the Institute of Anatomy, Center of Clinical Anatomy, Kiel University/Germany, from 2021 to 2024. Seven consecutive surgical training courses were evaluated. Prior to participation, all attendees were informed about the study and provided written informed consent. Data collection and processing were performed in compliance with the General Data Protection Regulation (GDPR) to ensure confidentiality and anonymity. The faculty and attendees agreed to the photodocumentation of the surgical training courses. The study was reported in accordance with the STROCSS 2021 guidelines for observational studies in surgery and required no registration, because patients were not involved [9].

### Didactic concept of surgical training courses

The surgical training courses were established in 2016 by a German working group of surgeons who aimed at standardizing the surgical technique for minimally invasive right hemicolectomy with CME by implementing the "critical view/open book" concept [8]. This approach pursued a twofold goal: First, before addressing the CME principle of central vascular ligation, each of the colonic segments has to be completely released from its embryological adhesions as initially emphasized by Hohenberger [1] to allow for complete resection of an intact mesocolon. This is achieved by the open book model in which each dissection plane is opened meticulously resulting in completely mobilized "pages" (retroperitoneal, ileocolic, transverse mesocolic, and mesogastric pages). Second, the surgical procedure is subdivided into well-defined critical views of safety to ensure maximal control during central vascular ligation and lymphadenectomy. The sequence of the critical views must be followed—failure to fully achieve a given critical view should prompt reevaluation of the surgical anatomy, correction of the dissection step or consideration of conversion to open surgery.

**Fig. 1:**
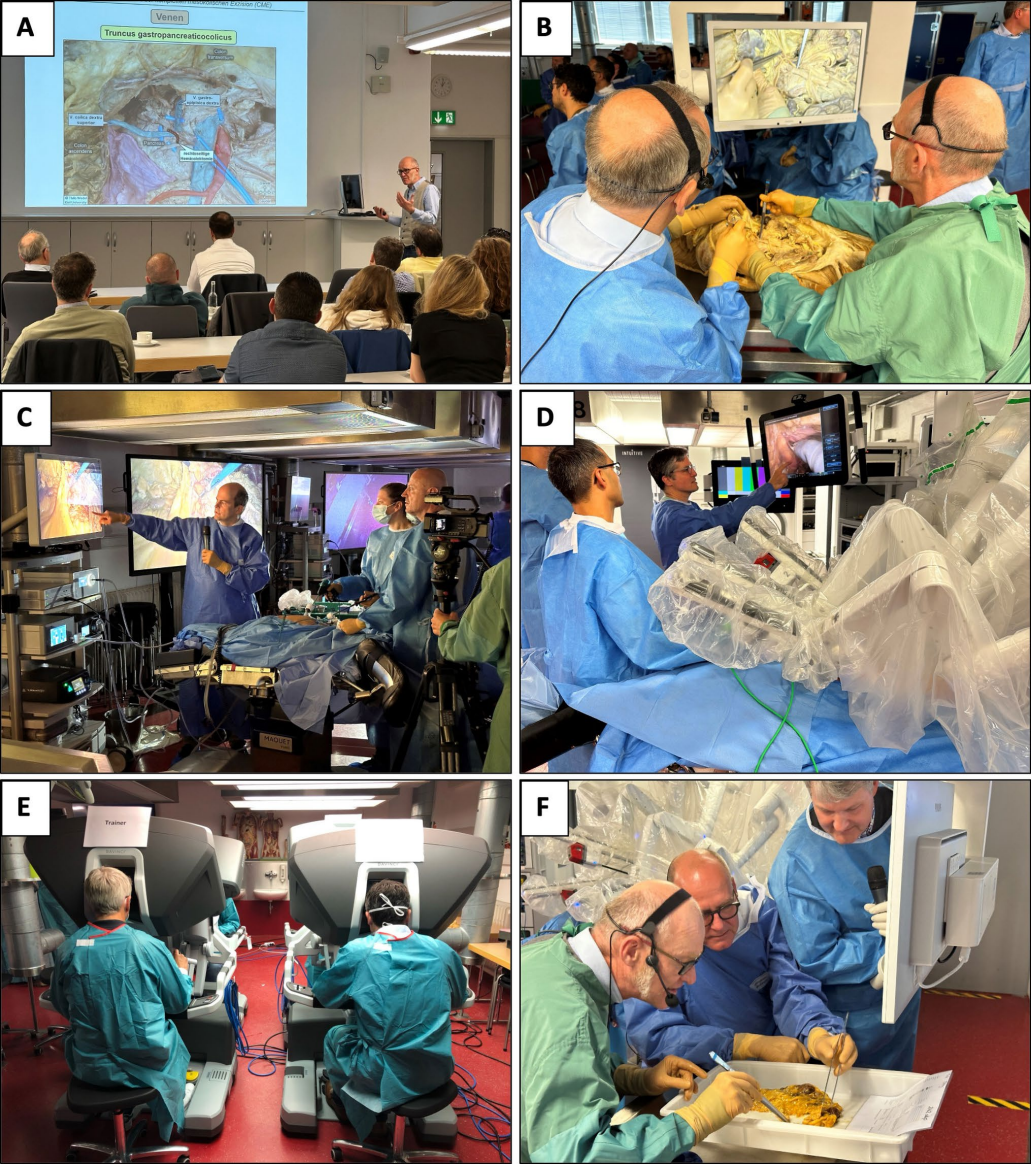
Teaching modules of the surgical training course. **A**: Anatomical and surgical lectures related to the "critical view/open book" concept. **B**: Demonstration of pre-dissected anatomical specimens. **C**: Laparoscopic working station. **D**: Robotic working station, patient cart. **E**: Dual robotic console. **F**: Quality assessment of resected specimens

The surgical training courses were designed for visceral surgeons specializing in laparoscopic or robotic colorectal surgery and were accredited by the German Society for General and Visceral Surgery (DGAV), Postgraduate Educational and Quality Center (WeiFoQ). The 2-day events were conducted twice annually and followed a structured four-stage training concept comprising the following modules (Fig. 1):

#### 1. Anatomical and surgical lectures

**Fig. 2:**
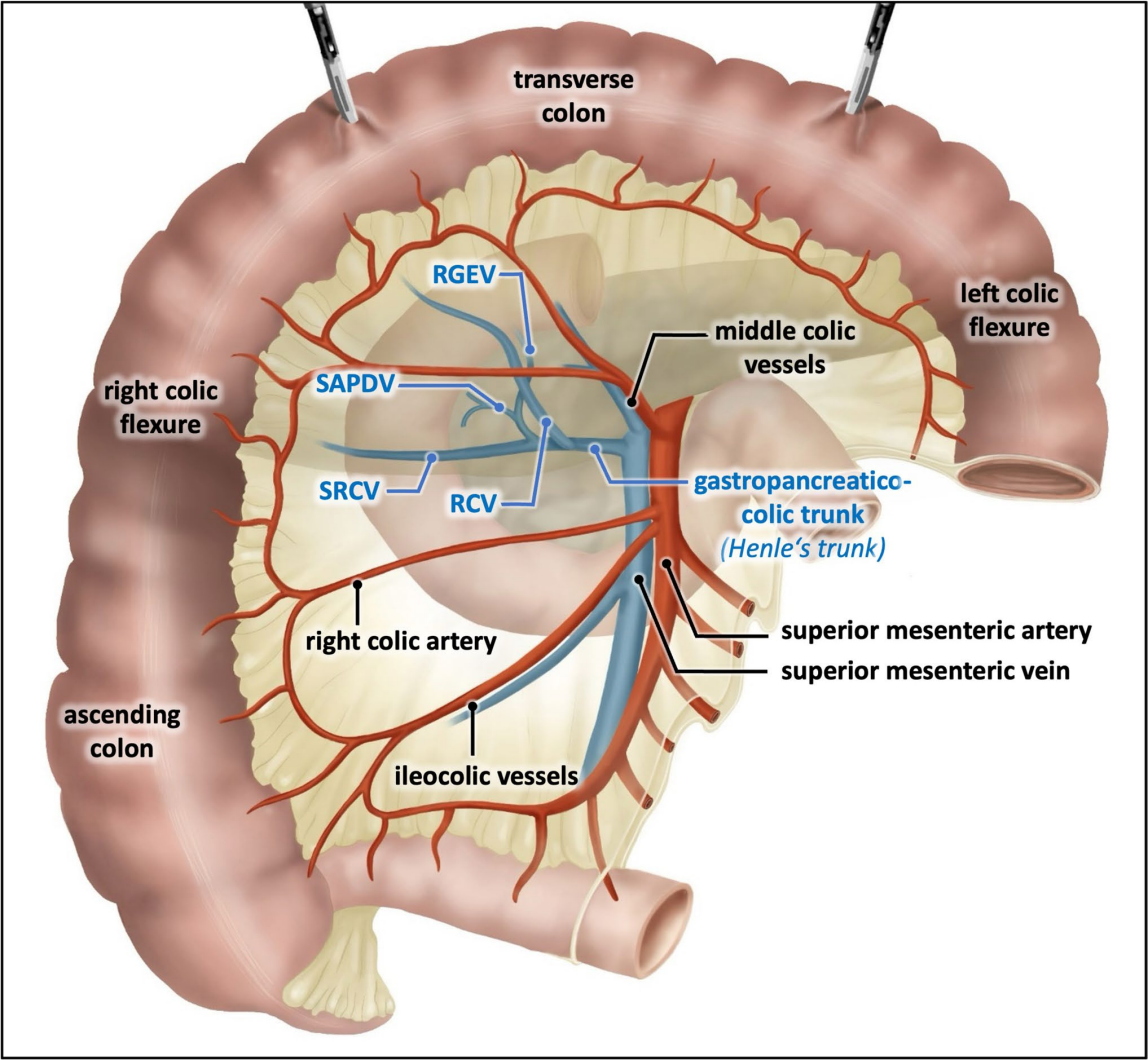
Overview of the vascular anatomy of the right colon. The transverse colon is retracted cranially; the duodenum and pancreas are visible behind the semi-transparent ascending and transverse mesocolon. Right colic vein (RCV), superior right colic vein (SRCV), right gastroepiploic vein (RGEV), superior anterior pancreaticoduodenal vein (SAPDV) drain into the gastropancreaticocolic trunk (Henle’s trunk). The large spectrum of vascular variations is not displayed in this standard illustration. Modified according to Heinze et al. [12]

The theoretical module involved lectures (30–45 min) supplemented by video clips (Fig. 1A) and covered the following topics:

- Rationale and principles of CME
- Surgical Anatomy (embryology, fascial planes, vascular variations, lymphatic drainage) related to CME (Fig. 2)
- "Critical view/open book" concept [8]
- Vascular mapping by preoperative CT angiography (AMIGO method) [10]
- Common pitfalls during minimally invasive CME
- Specific considerations in robotic CME

#### 2. Interactive demonstration of pre‑dissected anatomical specimens

Pre-dissected anatomical specimens were demonstrated jointly by an anatomist and a surgeon (Fig. 1B) to illustrate each critical view and the surgical dissection planes according to the "critical view/open book" concept. Special emphasis was given to the retrocolic fascial system [11] and the spectrum of vascular variations [12]. Key anatomical landmarks for each procedural step and structures at risk were highlighted and putative pitfalls were discussed. Following the demonstration, attendees were given the opportunity for hands-on studies of the demonstrated specimens to consolidate their anatomical and surgical understanding related to right hemicolectomy with CME.

#### 3. Surgical procedures on body donors

After introduction into the working stations (*n* = 6) minimally invasive right hemicolectomy with CME was performed in body donors (Fig. 1C–E). At each working station, three attendees jointly performed the procedure under the guidance of an experienced tutor following the “critical view/open book” concept. Each critical view had to be fully addressed, before proceeding to the next step to ensure standardization of the entire operation. In case of complex anatomical conditions or potential pitfalls, the procedure was paused and the surgical field was demonstrated and discussed with all attendees and faculty members.

Since 2022 the course setup has included four laparoscopic and two robotic working stations; prior to 2022 all procedures were performed laparoscopically. Each working station was equipped with a complete operating room setting including a mobile operating table (Yuno, Maquet Getinge Group, Rastatt, Germany), trolleys with laparoscopic equipment (Karl Storz, Tuttlingen, Germany), laparoscopic instruments (Karl Storz, Tuttlingen, Germany; Applied Medical, Rancho Santa Margarita, California), and surgical energy devices (Ethicon, Raritan, New Jersey; Applied Medical, Rancho Santa Margarita, California). The robotic stations (dual consoles) were equipped with a daVinci Xi system (Intuitive Surgical, Sunnyvale, California) coupled to an operating table with table motion (TruSystem 7000dV, Trumpf, Ditzingen, Germany) and a daVinci X system with a separate Maquet operating table.

#### 4. Evaluation of the resected specimen and postoperative surgical field

**Fig. 3:**
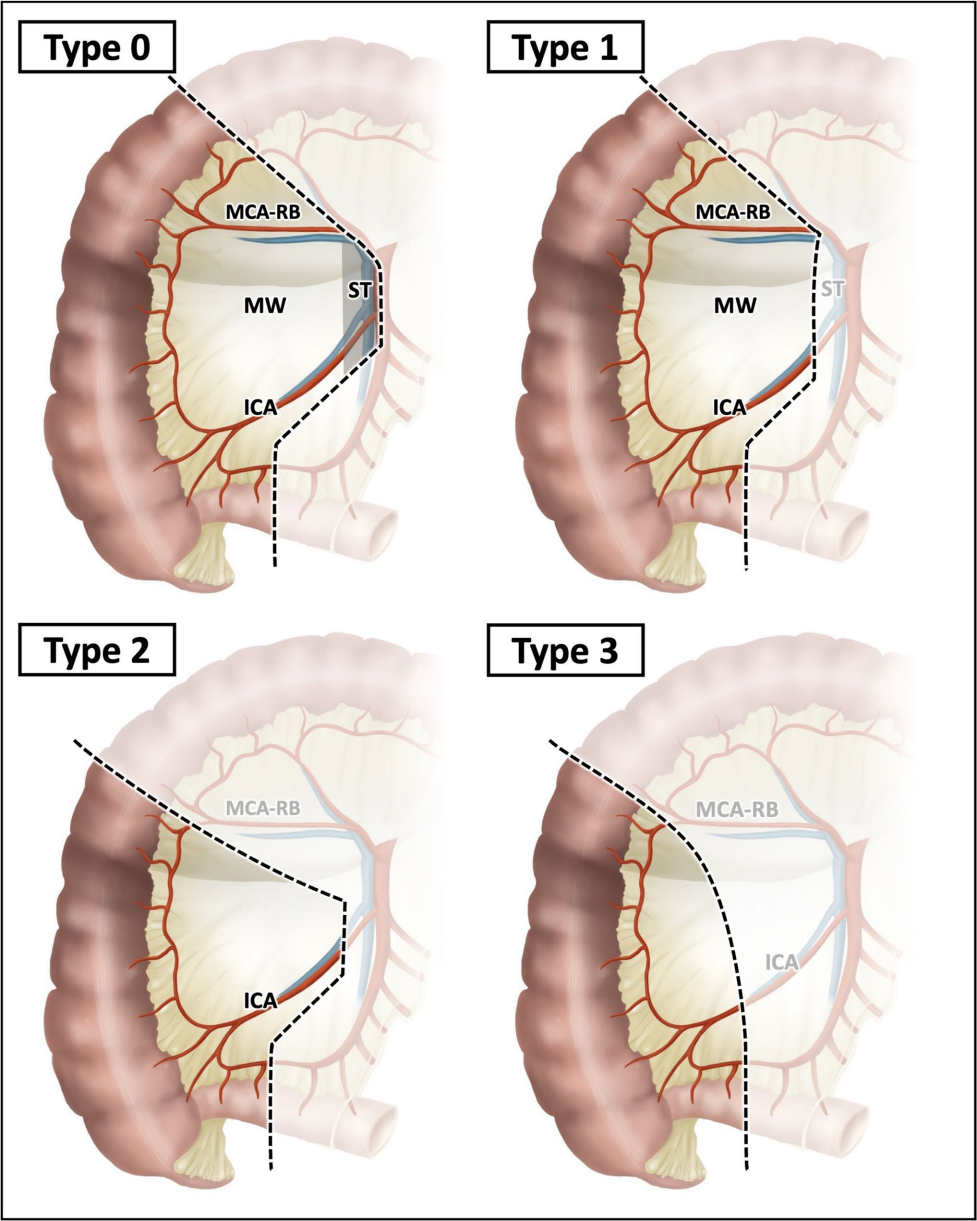
Classification system for CME in right-sided colon cancer adapted from Benz et al. [13] to assess the quality of resected specimens. Type 0: Ileocolic and middle colic pedicles are connected by a complete surgical trunk. Type 1: Ileocolic and middle colic pedicles are present, the surgical trunk is missing. Type 2: Most of the ileocolic pedicle is present (> 50%), the middle colic pedicle and the surgical trunk are missing. Type 3: Most of the ileocolic pedicle is missing (< 50%), the middle colic pedicle and the surgical trunk are missing. *ICA*, ileocolic artery, *MCA-RB*, middle colic artery, right branch, *MW*, mesocolic window

At the end of the surgical procedure, resected specimens were demonstrated to all attendees (Fig. 1F) and evaluated according to the classification system provided by Benz et al. (Fig. 3) [13]. The specimens were graded into the following types defined by decreasing completeness and integrity of the mesocolon (type 0 → type 3):

- Type 0: ileocolic and middle colic pedicles connected by a complete surgical trunk
- Type 1: ileocolic and middle colic pedicles present, surgical trunk missing
- Type 2: ileocolic pedicle partly present [> 50%], middle colic pedicle and surgical trunk missing
- Type 3: ileocolic pedicle [< 50%], middle colic pedicle and surgical trunk missing

The macroscopic integrity was graded into subcategories a (intact mesocolon), b (laceration of mesocolon), c (laceration of mesocolon reaching the bowel wall next to the tumor). High-resolution photographs of all resected specimens (ventral and dorsal aspects) were taken to document the specimen quality (Sony Alpha 7 III, Sony FE 90 mm F2.8, Japan).

Moreover, the postoperative surgical field of each working station was discussed in detail related to the following aspects: (1) confirmation of the correct surgical dissection planes, (2) confirmation of central vascular ligation and report of the individual topography of right-sided colic arteries and veins including the specific configuration of the gastropancreaticocolic trunk/Henle’s trunk, (3) report of case-related technical difficulties and subsequent surgical management. The quality control of the resected specimen and of the postoperative surgical field adhered to the recommendations for the "assessment of performance" provided by the consensus statements on CME [6].

### Body donors used in surgical training courses

Body donors were recruited from the local body donation program (Institute of Anatomy, Kiel University, Germany) after previous informed written consent to be used for medical research and educational purposes and approval by the local ethics committee (D514/24, Medical Faculty, Kiel University). Body donors with previous diseases and surgeries involving the abdomen were excluded. For surgical interventions, 36 body donors (21 males, 15 females) with a mean age of 82 years (57–94 years) were fixed by a solution containing ethanol (70%) and glycerin (30%) via femoral arteries allowing for minimally invasive surgery due to a true-to-life consistency of tissues [14]. For pre-dissected anatomical specimens, fixation was performed by a solution containing formaldehyde (3%), ethanol (75%), and glycerin (7%) via femoral arteries and subsequent preservation by immersion in ethanol (70%).

### Evaluation of surgical training courses

The evaluation of the surgical training courses was conducted two weeks post-training using an anonymous GDPR-compliant online survey (LimeSurvey, Hamburg, Germany). Attendees completed a structured questionnaire consisting of 17 questions divided into three sections: (1) professional background and training level, (2) previous surgical experience with right hemicolectomy and CME, (3) newly gained anatomical and surgical insights related to CME based on the “critical view/open book” concept, its application in the surgical training course and its adoption into clinical practice (Suppl. 1).

### Statistical analysis

Statistical analysis was performed using SPSS 29.0 (IBM Company, Armonk, NY, USA). The Shapiro–Wilk test was used to test for distribution of data. Differences between the laparoscopic and robotic groups related to their answer behavior and quality of resected specimens were analyzed by using the Mann–Whitney-*U* test. The significance level was set at 5% (*p* < 0.05).

## Results

### Professional background of attendees

From 2021 to 2024, seven surgical training courses were evaluated involving 125 attendees with a response rate of 68% (*n* = 85). The majority of respondents (75.3%) had more than 10 years of experience in visceral surgery, 14.1% reported 7–9 years, 3.5% reported 4–6 years, and 5.9% reported less than 4 years. Most attendees were affiliated with secondary care hospitals (34.1%), followed by community hospitals (27.1%), university hospitals (22.4%), and tertiary care hospitals (16.5%). Regarding their position and level of training, 81.2% of attendees were senior surgeons, 10.6% were board-certified consultants, 4.7% were heads of department, and 3.5% were residents in surgical training (Table 1).

**Table 1:** Professional background of attendees

Experience in visceral surgery	≥ 10 years	75.3%
	7–9 years	14.1%
	4–6 years	3.5%
	0–3 years	5.9%
Affiliated hospitals	University hospital	22.4%
	Tertiary care hospital	16.5%
	Secondary care hospital	34.1%
	Community hospital	27.1%
Position/level of training	Head of department	4.7%
	Senior attending surgeon	81.2%
	Board-certified consultant	10.6%
	Resident in surgical training	3.5%

### Prior experience with right hemicolectomy

Before attending the surgical training course, 65.9% of attendees had performed right hemicolectomy with CME as primary surgeon via open approach, 42.4% laparoscopically, and 15.3% robot-assisted. Surgical assistance was slightly more common, with 72.9% having assisted in open, 55.3% in laparoscopic, and 29.4% in robotic right hemicolectomy with CME. Right hemicolectomy without CME had been performed by 81.2% of attendees via open approach, 65.9% laparoscopically, and 12.9% robot-assisted. Notably, all attendees had performed a right hemicolectomy in some form prior to the surgical training course.

### Evaluation of the surgical training course

#### New anatomical insights

**Fig. 4:**
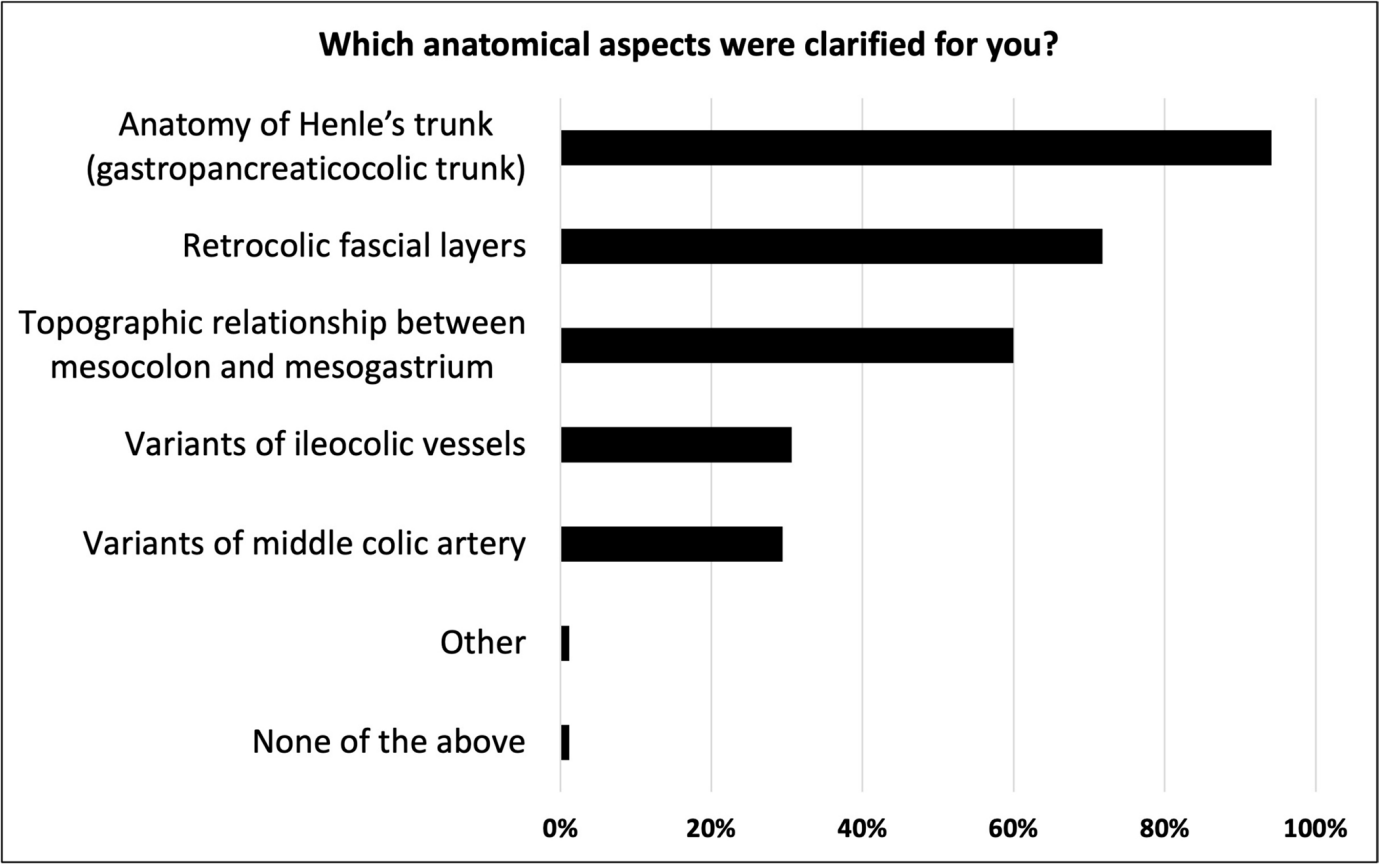
New anatomical insights gained from surgical training courses

94.1% of attendees reported an improved understanding of the anatomy of the gastropancreaticocolic trunk (Henle’s trunk) and its variations. Further anatomical insights were gained in regard to the retrocolic fascial system (71.8%), the topographical relationship between the mesocolon and mesogastrium (60.0%), and the different trajectories of both ileocolic vessels (30.6%) and the middle colic artery (29.4%) (Fig. 4). In free-text comments, the large spectrum of vascular variations of the right colon was reported as a novel aspect.

#### New procedural insights

**Fig. 5:**
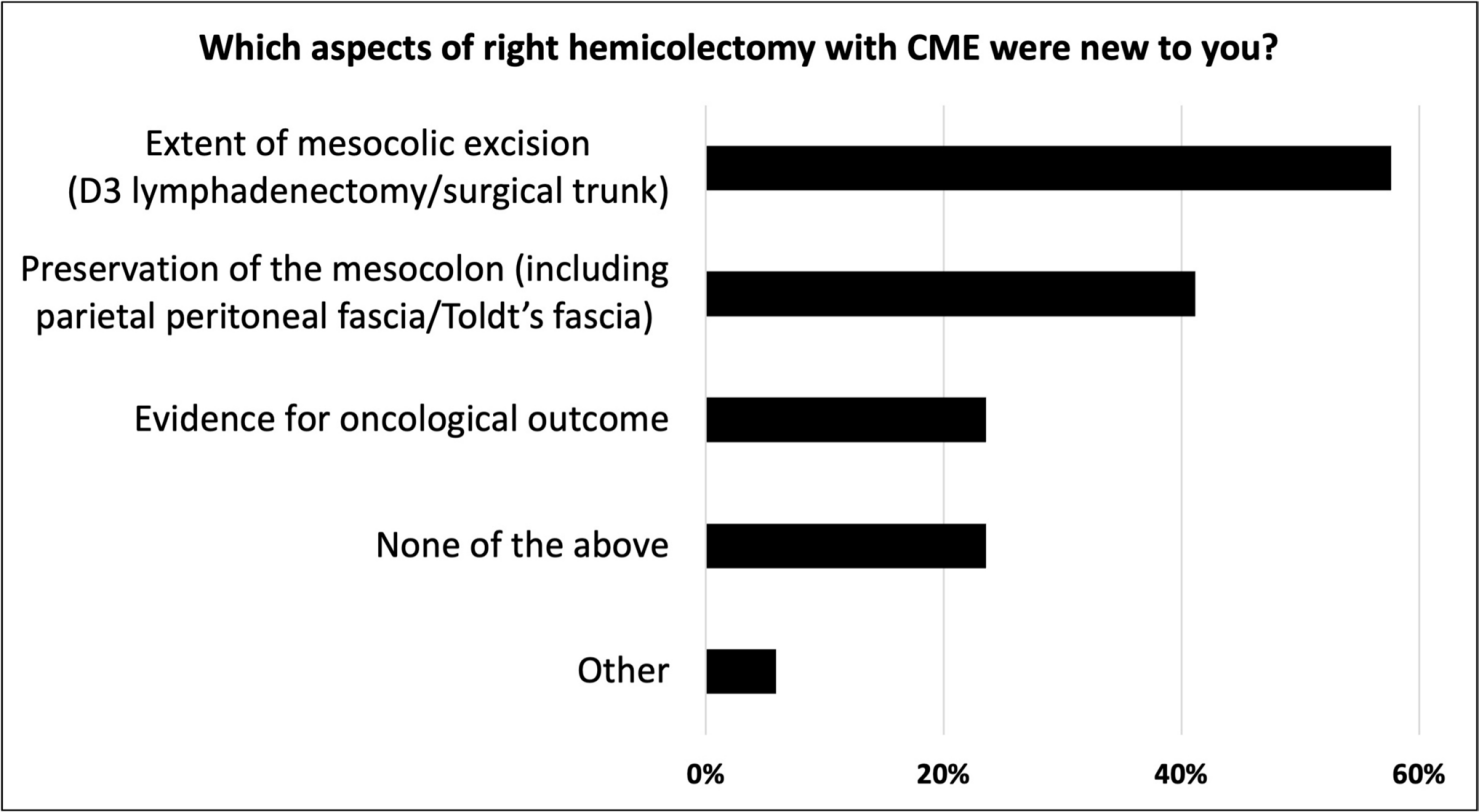
New procedural insights gained from surgical training courses

57.6% of attendees reported a better understanding of the required extent of mesocolic excision including the exposure of the surgical trunk to achieve a D3-lymphadenectomy. Preservation of the mesocolon and the parietal peritoneal fascia (Toldt’s fascia) at the dorsal aspect of the specimen was regarded as a novel aspect by 41.2%. Furthermore, 23.5% of attendees gained new insights into the oncological rationale and scientific evidence for CME (Fig. 5). In addition, the surgical management of the gastropancreaticocolic trunk (Henle’s trunk) was highlighted as a novel aspect in free-text responses.

### Applicability and usefulness of the “critical view/open book" concept

**Fig. 6:**
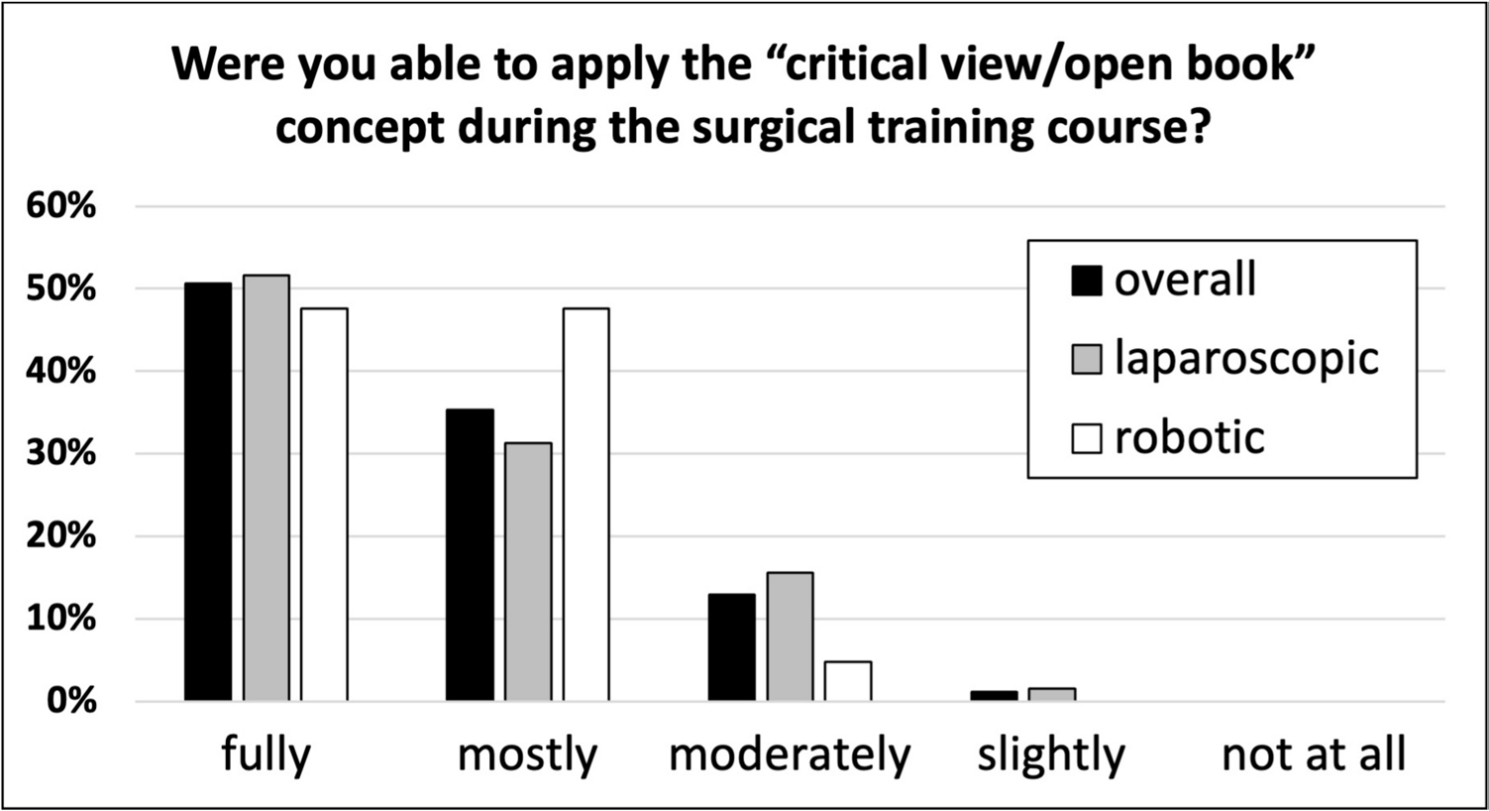
Applicability of the “critical view/open book” concept during surgical training courses. Differences between the laparoscopic and robotic groups were not significant

The "critical view/open book" concept could be fully applied by 50.6% of attendees and mostly applied by 35.3% during the surgical training course. 12.9% reported moderate and 1.2% reported limited applicability. No statistically significant difference was found between the laparoscopic and robotic training groups (Fig. 6).

**Fig. 7:**
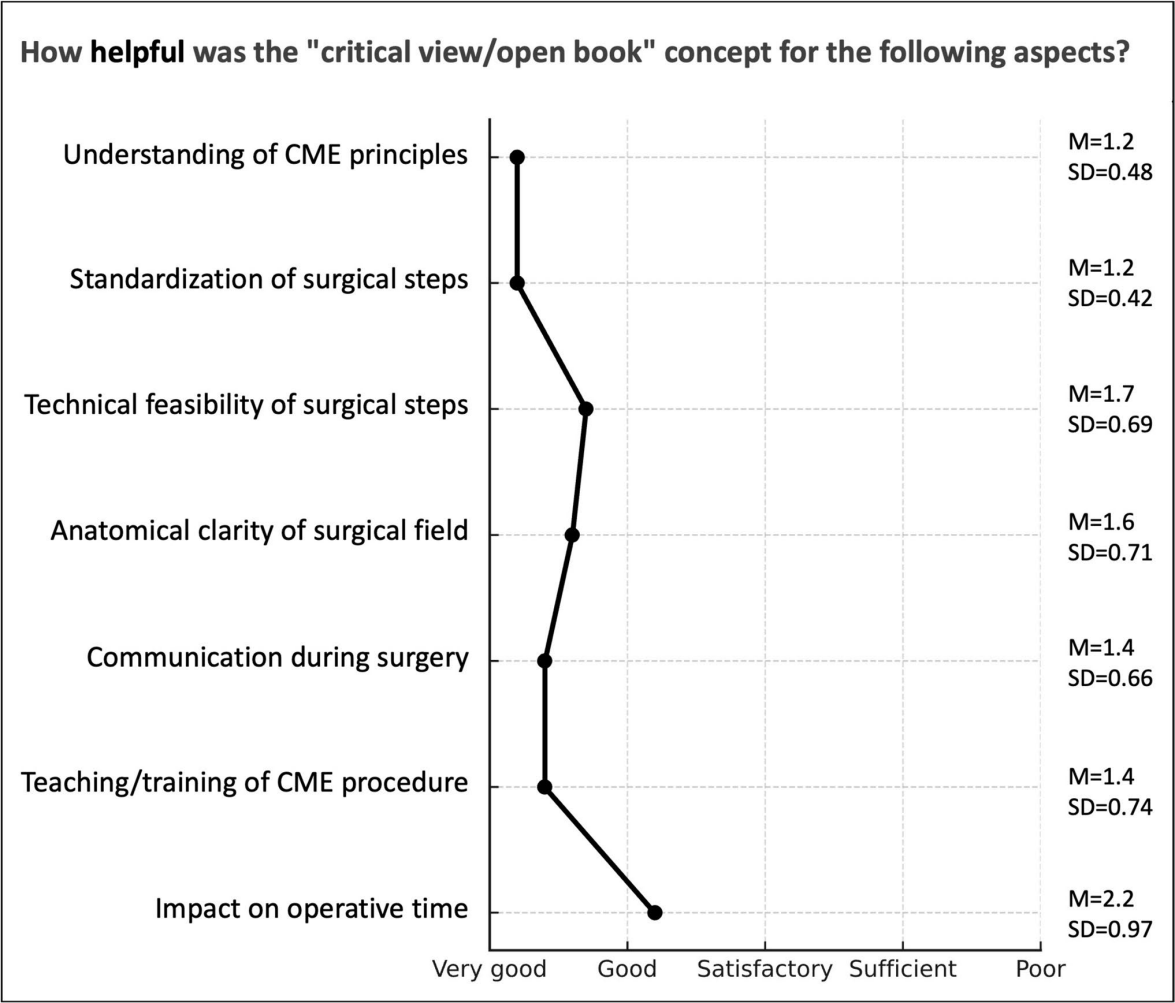
Usefulness of the "critical view/open book" concept

The usefulness of the "critical view/open book" concept for various surgical aspects was assessed using a five-point grading scale/Likert scale (1 = very good, 5 = poor) (Fig. 7). Attendees rated the concept highest for its “standardization of surgical steps” and its contribution to the “understanding of CME principles” (mean grade: 1.2). “Communication during surgery” and the “teaching/training suitability of CME” were also evaluated positively (1.4), followed by “anatomical clarity of the surgical field” (1.6) and “technical feasibility of surgical steps” (1.7). The lowest rating was given for the “impact on operative time” (2.2).

These ratings were supported by free-text comments: Attendees highlighted a perceived increase in procedural safety (*n* = 6) and oncological radicality (*n* = 6). A few participants also appreciated the embryologically coherent dissection strategy (*n* = 2) (Table 2). Critical remarks focused primarily on the extended operative time required for certain procedural steps, such as repositioning the small bowel for optimal exposure or repeated adjustments of the operating table position (*n* = 6). Further concerns included potential complications such as vascular or pancreatic injury (*n* = 4), doubts regarding the oncological benefit in relation to the invasiveness of the procedure (*n* = 4), and the prolonged learning curve for less experienced surgeons (*n* = 1) (Table 3).

**Table 2:** Positive free-text responses regarding the "critical view/open book" concept

What did you like most about the "critical view/open book" concept?	
Aspects	Frequency
Structured and standardized surgical approach	33
Improved anatomical orientation and clarity	25
Increased procedural safety	6
Enhanced oncological radicality	6
Improved comprehensibility and teachability of the procedure	3
Embryologically coherent surgical approach	2

**Table 3:** Critical free-text responses regarding the "critical view/open book" concept

What did you dislike about the "critical view/open book" concept?	
Aspects	Frequency
Prolonged operative time (e.g., repositioning small bowel/patient)	6
Concerns about potential complications (e.g., vascular/pancreatic injury)	4
Doubts regarding oncological benefit relative to invasiveness of the procedure	4
Prolonged learning curve for less experienced surgeons	1

### Quality assessment of resected specimens

**Fig. 8:**
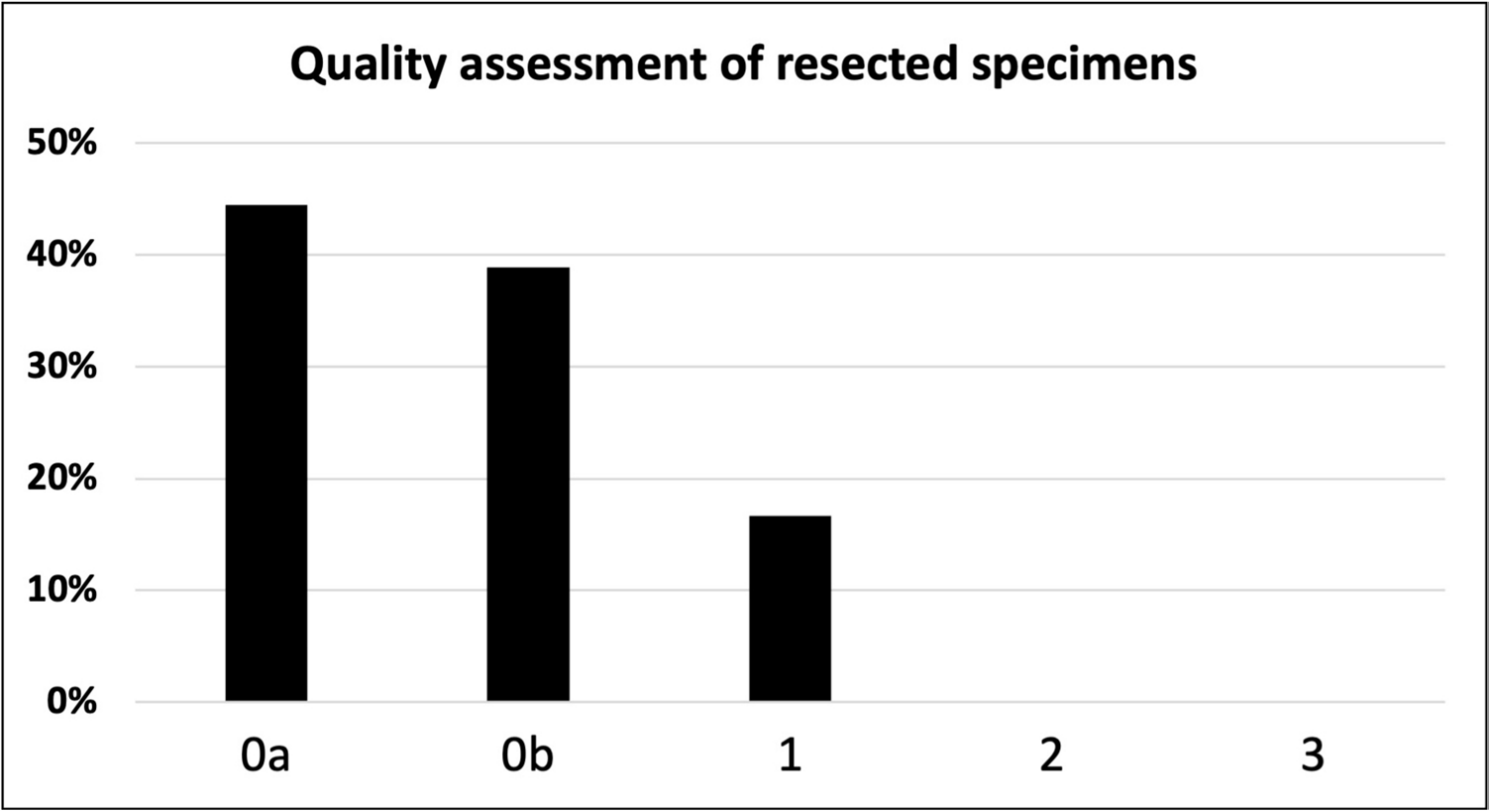
Results of the quality assessment of resected specimens according to the classification system provided by Benz et al. [13]

**Fig. 9:**
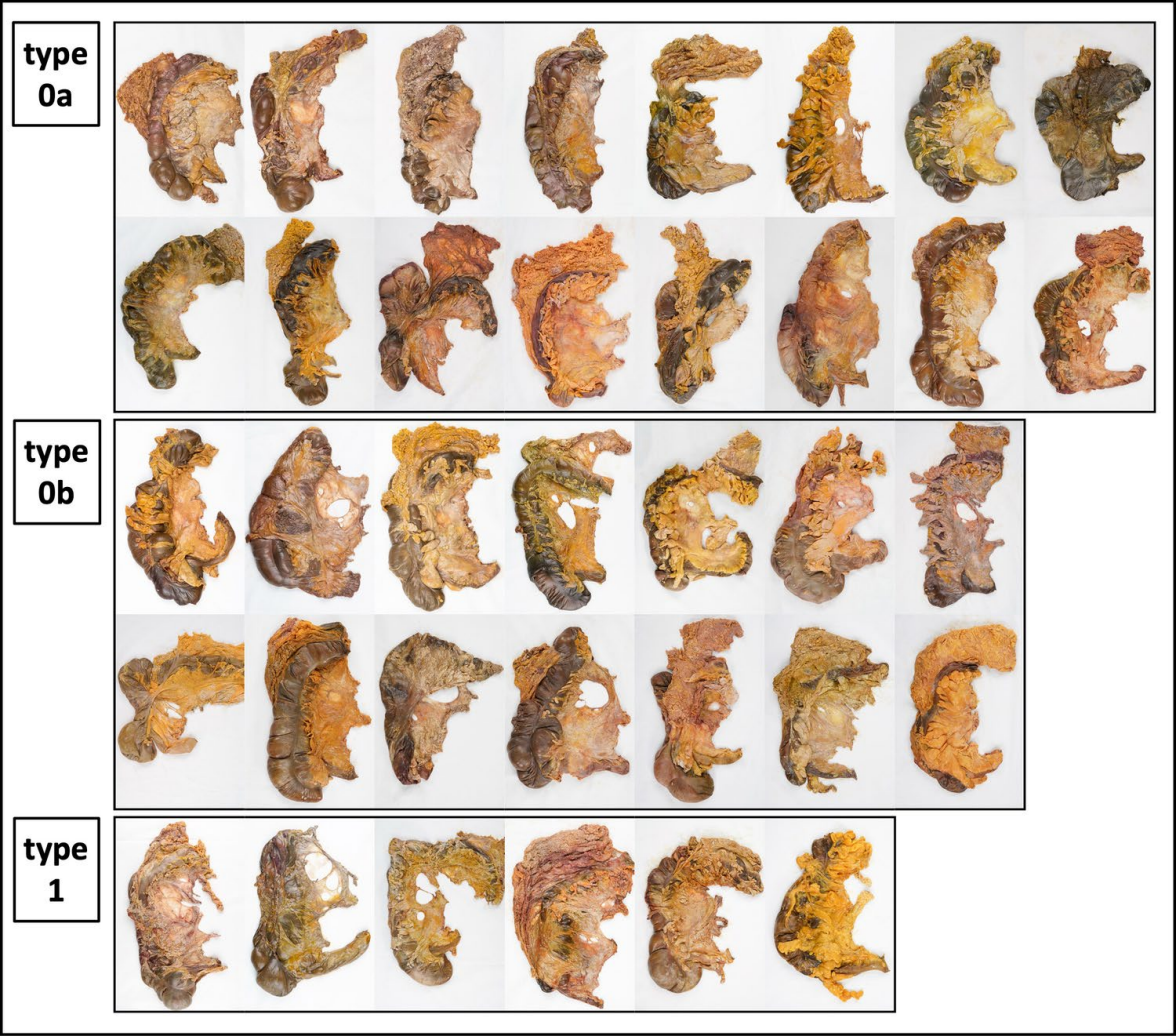
Photodocumentation of all resected specimens harvested during the surgical training courses (2021–2024) and ordered by achieved resection quality (classification type)

**Fig. 10:**
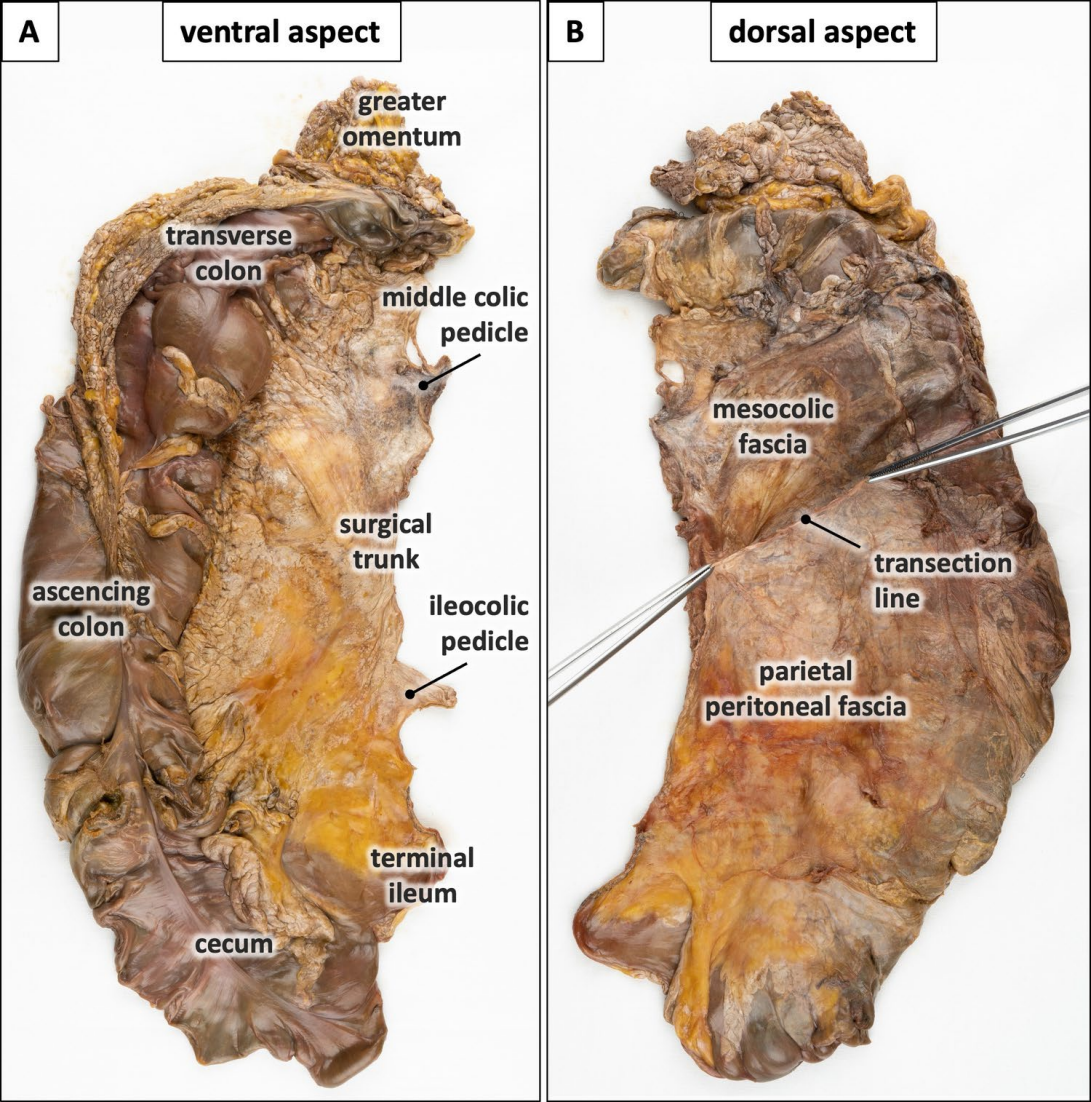
Representative specimen harvested during the surgical training course classified as type 0a (ileocolic and middle colic pedicles are connected by a complete surgical trunk). **A**: Ventral aspect. **B**: Dorsal aspect displaying the transection line of the parietal peritoneal fascia (Toldt’s fascia) indicated by two forceps

A total of 36 harvested specimens (26 by laparoscopic surgery, 8 by robotic surgery) were evaluated according to the classification system provided by Benz et al. [13]. Overall, 44.4% of specimens were classified as type 0a, 38.9% as type 0b, and 16.7% as type 1 (Figs. 8, 9, and 10). No specimens were assigned to type 2 or type 3. No statistically significant difference between the laparoscopic and robotic groups was found.

### Adoption of the "critical view/open book" concept

**Fig. 11:**
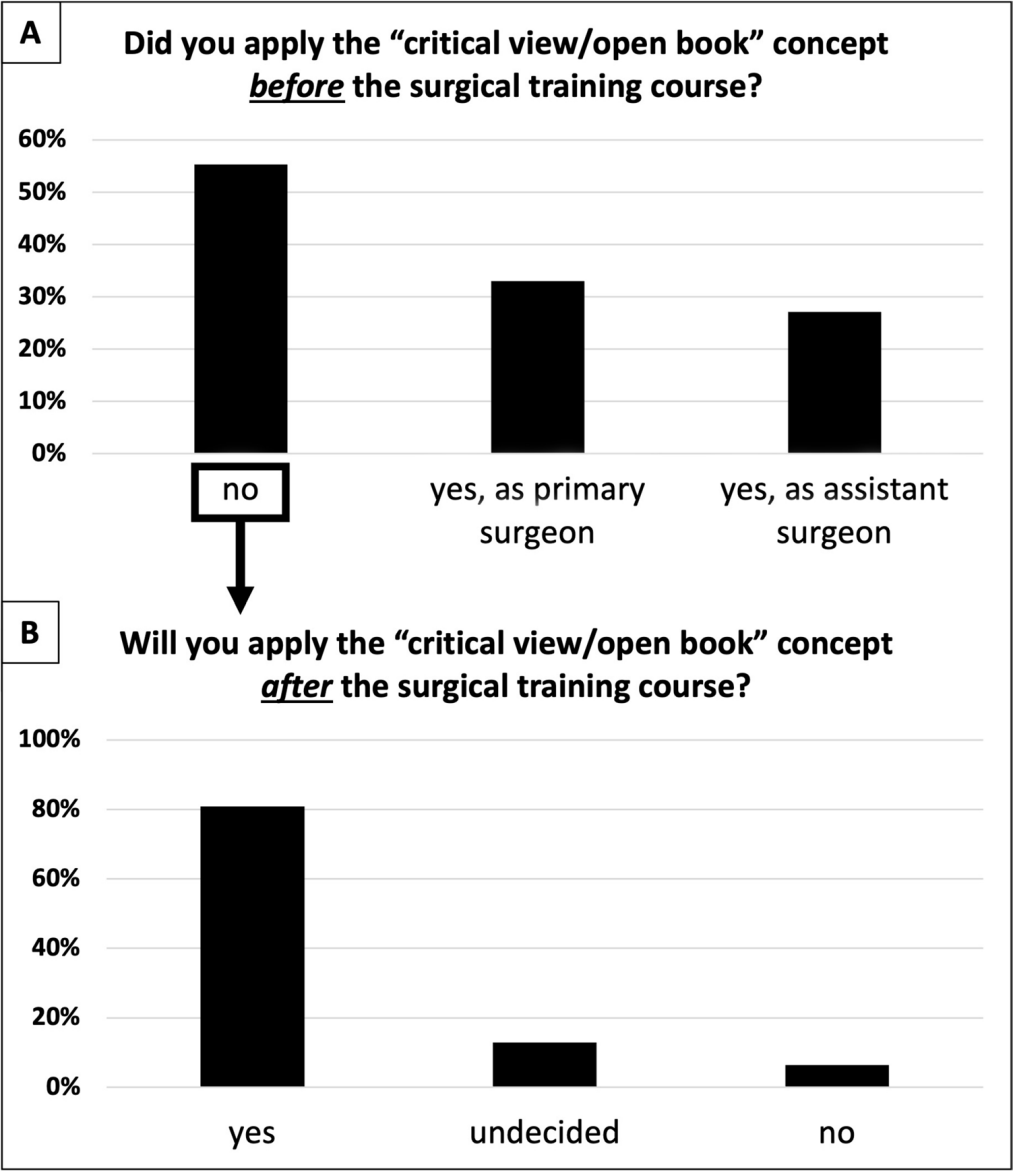
**A**: Application of the "critical view/open book" concept before the surgical training course. **B**: Adoption of the "critical view/open book" concept into one’s own surgical practice after the surgical training course

Before the surgical training course, 32.9% of attendees reported having applied the "critical view/open book" concept as primary surgeon and 27.1% as assistant surgeon, while 55.3% had not used this concept in their surgical practice at all (Fig. 11A). Among those who had not previously applied the "critical view/open book" concept, 80.9% stated that they have implemented or are planning to implement this surgical approach after the completion of the surgical training course (Fig. 11B). 12.8% of attendees were still undecided, while 6.4% reported no intention to apply the concept. In free-text responses, reluctance to adopt the "critical view/open book" concept was primarily attributed to the increased operative time and a lack of institutional or supervisory support.

## Discussion

### Surgical anatomy related to CME—an essential prerequisite

Failure to achieve a successful right hemicolectomy with CME is often due to an incomplete understanding of the complex anatomy. Among the novel anatomical insights gained by the attendees during the surgical training courses the most relevant issues concerned the topography of the retrocolic fascial layers and the rather broad spectrum of vascular variations of both arteries and veins including the gastropancreaticocolic trunk (Henle’s trunk). The interest in these anatomical topics is well reflected by an impressively large number of reviews and studies which have addressed and revisited the anatomy of mesocolic and retrocolic fasciae [11, 15–23] and the vascular supply of the right colon [12, 24–31]. In their systematic review and meta-analysis Cirocchi et al. suggested that the variants of the colic vascular supply are "the Achilles heel of right hemicolectomy with CME" [23]. This notion is well in line with the recommendations for an optimal training curriculum for CME derived from a consensus report in which "anatomy teaching" received the highest agreement (97%) and "appraisal of the anatomy of the right colon, including the vascular supply" was explicitly emphasized [6].

### Surgical training on body donors—the most realistic scenario

The surgical procedures were carried out on body donors instead of using virtual reality simulators or artificial anatomical models. This decision was primarily based on a previous survey among surgeons who reported a high level of satisfaction regarding the realistic tissue and organ properties and the technical feasibility of laparoscopic procedures on body donors fixed by ethanol and glycerin [14]. When compared to virtual reality simulations, body donors were perceived as a significantly better model for laparoscopic training, while simulators may still be useful when training junior trainees in basic laparoscopic tasks [32]. Similar conclusions were drawn from a study performed by an international Colorectal Surgery Training Group, who compared body donors and augmented reality simulator models for straight laparoscopic colorectal skills acquisition training [33]: The main drawbacks of simulators consisted in a poorer replication of human anatomy, tissue planes, and tissue consistency—all of these issues are of utmost importance to perform complex surgical procedures such as CME. Accordingly, in the consensus on the training framework for CME [6], the use of body donors has been regarded as the preferred option for hands-on surgical training courses and has yielded an agreement of 83% [7].

Nevertheless, due to cost-efficiency reasons and unhindered accessibility, surgical training courses using body donors should be provided by centralized and experienced facilities offering postgraduate educational programs at an international level. Moreover, soft-tissue fixation protocols for body donors [34] should be preferred over fresh-frozen preservation allowing resource-saving multiple surgical procedures in several body regions.

Recently, an anatomically true-to-life phantom based on 3D printing and medical imaging [35] and a laparoscopic right hemicolectomy training simulator (COLOMASTER) based on polyester fibers and hydrogel [36] have been developed for CME training purposes. These simulators become increasingly important as complementary tools for efficient off-the-job training and assessment of basic surgical competency and should be provided prior to surgical training courses based on body donors. Moreover, a modular training course design in which the theoretical modules are provided as e-learning pre-course content may further improve cost-efficiency and accessibility.

### Applicability and usefulness of the "critical view/open book" concept—pros and cons

The "critical view/open book" concept could be fully/mostly applied by 85.9% of the attendees during the surgical training course, while only 14.1% reported moderate or slight applicability. This rather high acceptance rate was primarily due to a deepened understanding of the CME principles and the segmentation of the complex surgical procedure into well-defined surgical steps. Obviously, the standardization achieved by the critical views of safety was helpful in focusing on clearly described tasks.

In fact, the need for standardization of CME surgery has been increasingly advocated in the current literature: Gomez et al. deconstructed robotic right hemicolectomy with CME and intracorporeal anastomosis into performance metrics (15 procedure phases with 125 steps, 150 errors, and 139 critical errors) and considered these metrics imperative for the development of a safe and structured training for robotic CME [37]. Similarly, an international expert consensus (RRoC-STAR, Radical Right Colectomy—Surgical Technique Approved Report) provided 20 items for standardized terminology, reporting and defining surgical steps for right hemicolectomy to enable data homogenization from current and future studies [38]. In line with these structured guidelines our survey also confirmed that the "critical view/open book" concept facilitated the unequivocal communication between surgeons during the intervention and the ability to properly teach and train the procedure by breaking it down into manageable steps.

In regard to the different surgical approaches for CME (e.g., lateral, medial, cranial, caudal, or combined approach) a recent review emphasized that regardless of the chosen approach "the key point is entering Toldt´s space quickly and accurately. If the correct plane is not entered, the mesenteric vessels may be damaged (…)" [39]. This recommendation is well in accordance with embryological considerations advocated by Matsuda et al. who pointed out that reversal of the complex torsion and fusion of the right colon/mesocolon should be performed prior to central vascular ligation [40]. The “open-book/critical-view” concept addresses explicitly these considerations by first detaching the mesocolon from its fusion to underlying structures, before focusing on the identification and ligation of its vascular supply. This technical strategy facilitates the harvesting of an intact mesocolon (in particular its dorsal aspect), the integrity of the duodenum and pancreas as well as the control of vascular pedicles during central ligation, as suggested by previous studies [41–47].

The major drawbacks of the “open-book/critical-view” concept compared to conventional right hemicolectomy mentioned by the attendees were the extended operative time, the invasiveness of the procedure, and the prolonged learning curve. Although these aspects are valid and may deter the application of the “open-book/critical-view” concept, they are inherent to a properly performed CME with central vascular ligation and adequate lymphadenectomy regardless of the surgical approaches and guidelines applied. Subsequently, teaching and training curricula should be provided to overcome these restraints.

### High quality of resected specimens following the "critical view/open book" concept

One of the most encouraging results of the study was the high quality of resected specimens after execution of the standardized critical views. 83.3% of specimens fulfilled the criteria for type 0 (ileocolic and middle colic pedicles connected by a complete surgical trunk), only 16.7% were assigned to type 1 (ileocolic and middle colic pedicles present, surgical trunk missing), while type 2 and 3 were not encountered. This level of performance was rather unexpected, as only one third of attendees reported having applied the "critical view/open book" concept before, whereas two thirds of attendees had no previous experience with this type of surgery as primary surgeon. Admittedly, although the quality of the resected specimen is considered to be a direct indicator for a proper CME, it is a surrogate parameter depending on multiple variables not exclusively linked to the application of the "critical view/open book" concept or individual surgical proficiency. Thus, the quality of the resected specimens may also reflect the supervision and controlled environment.

### Adoption of the "critical view/open book" concept

After having completed the surgical training course, the majority of attendees (80.9%) not previously experienced with the "critical view/open book" concept were convinced to implement this surgical approach in their clinical routine. Obviously, the advantages of this concept related to anatomical clarity, technical feasibility, and retrieved specimen quality outweighed the concerns. However, the intention to adopt the "critical view/open book" concept is more aspirational than observational—its implementation and clinical impact have to be monitored in follow-up studies.

Reluctance to adopt the "critical view/open book" concept was primarily due to logistic aspects (increased operative time, lack of institutional/supervisory support). These issues typically reflect the situation in which health care providers are not able (or willing) to promote a required surgical paradigm shift due to economic or personal reasons.

### Limitations of the study

The survey is limited by an incomplete response rate (two thirds of attendees) and a heterogeneously composed collective (surgeons with different experiences and positions). However, regardless of their surgical background and provenience, attendees had participated in the surgical training courses, because they were genuinely interested and intrinsically motivated to familiarize themselves with the surgical management of CME based on the "critical view/open book" concept.

The preponderance of senior surgeons may constitute a selection bias explaining the rather good acceptance of the "critical view/open book" concept and the convincing specimen quality. On the other hand, only 42.4% had performed right hemicolectomy with CME laparoscopically and 15.3% robot-assisted prior to the surgical training course. Given the technical challenges of minimally invasive CME, a structured and standardized training of this procedure is also reasonable even for experienced surgeons and may facilitate broader institutional adoption of this concept by providing a favorable teaching environment for junior surgeons. Furthermore, the educational value for experienced surgeons is substantiated by reported new anatomical and surgical insights gained during the surgical training course regardless of prior surgical experience.

The logistic setting (e.g., limited time frame, three attendees per working station, differing conditions of body donors) and the didactic focus (e.g., meticulous execution and repeated practice of procedural steps, interactive demonstration of anatomical variations and surgical pitfalls) of the surgical training courses did not allow full recording of individual performance metrics such as those proposed by an international expert consensus (15 procedure phases with 125 steps) [37]. While the adherence to the critical views and intraoperative errors were monitored at each working station, other objective measures (e.g., GEARS-like scoring, time to achieve each critical view) were not systematically evaluated.

Although a potential bias was the introduction of robotic working stations in addition to laparoscopic working stations during the study, no statistically significant differences could be observed between these two groups in regard to the applicability of the "critical view/open book" concept. Likewise, the quality of resected specimens showed no statistically significant difference between the two groups. However, the small number of robotic specimens limits the interpretability of this comparison to preliminary descriptive evidence.

## Conclusion

In their review on "Implementing complete mesocolic excision for colon cancer – mission completed?" Croner & Hohenberger have emphasized that "teaching programs are needed for minimally invasive CME to facilitate this technique in the same quality compared to open surgery" [48]. We believe that the setting of surgical training courses described in this study contributes substantially to achieving these aims targeted by the inaugurators of CME. The "critical view/open book" concept provides not only essential anatomical insights into the principles of CME but also a feasible step-by-step procedure for minimally invasive CME as evidenced by the high quality of resected specimens. Thus, we consider this type of surgical training course to be a promising teaching module that may contribute to the broader implementation of minimally invasive CME as the standard procedure of surgical care.

## Supplementary Information

Below is the link to the electronic supplementary material.

Supplementary file1 (DOCX 26 KB)

## Data Availability

Data available on request from the authors.
