# Supplementary material for: Efficacy of the "critical view/open book" concept for implementing standardized right hemicolectomy with complete mesocolic excision—results from a series of minimally invasive surgical training courses
Source: Surg Endosc. 2026 May 29;40(7):6159–75. doi: 10.1007/s00464-026-12901-7 (PMC13369770; doi:10.1007/s00464-026-12901-7)
Supplement: Supplementary file 1 — Supplementary file1 (DOCX 26 KB) [file 464_2026_12901_MOESM1_ESM.docx]

**Supplementary Material**

**Supplementary Table 1: Questionnaire**

| **Current professional status** | | |
| --- | --- | --- |
| Question 1) | Which type of hospital are you affiliated with? | - Community hospital - Secondary care hospital - Tertiary care hospital - University hospital |
| Question 2) | What is your current position at your institution? | - Resident in surgical training - Board-certified consultant - Senior attending surgeon - Head of department |
| Question 3) | How many years of experience do you have in visceral surgery? | - 0–3 years - 4–6 years - 7–9 years - ≥ 10 years |
| **Previous surgical experience in oncologic right hemicolectomy** | | |
| Question 4) | Which of the following surgical approaches have you assisted prior to course participation?  *[Multiple answers possible]* | - Open surgery without CME - Open surgery with CME - Laparoscopic surgery without CME - Laparoscopic surgery with CME - Robot-assisted surgery without CME - Robot-assisted surgery with CME - No prior assistance |
| Question 5) | Which of the following surgical approaches have you performed as primary surgeon prior to course participation?  *[Multiple answers possible]* | - Open surgery without CME - Open surgery with CME - Laparoscopic surgery without CME - Laparoscopic surgery with CME - Robot-assisted surgery without CME - Robot-assisted surgery with CME - No prior experience as primary surgeon |
| Question 6) | Did you apply the “critical view/open book” concept *before* the surgical training course?  *[Multiple answers possible]* | - No - Yes, as primary surgeon - Yes, as assistant surgeon |
| **“Critical view/open book” concept in the surgical training course** | | |
| Question 7) | Which aspects of right hemicolectomy with CME were new to you?  *[Multiple answers possible]* | - Evidence for oncological outcome - Extent of mesocolic excision (D3 lymphadenectomy/surgical trunk) - Preservation of the mesocolon (including parietal peritoneal fascia/Toldt’s fascia) - None of the above - Other: free-text response |
| Question 8) | Which anatomical aspects were clarified for you?  *[Multiple answers possible]* | - Retrocolic fascial layers - Variants of ileocolic vessels - Variants of middle colic artery - Anatomy of Henle’s trunk (gastropancreaticocolic trunk) - Topographic relationship between mesocolon and mesogastrium - None of the above - Other: free-text response |
| Question 9) | To what extent were you able to apply the “critical view/open book” concept during the surgical training course? | - Fully - Mostly - Moderately - Slightly - Not at all |
| **“Critical view/open book” concept in clinical practice** | | |
| Question 10) | How helpful was the “critical view/open book” concept for the following aspects?  *[Please rate using a five-point grading scale]*   - Understanding of CME principles - Standardization of surgical steps - Technical feasibility of surgical steps - Anatomical clarity of surgical field - Communication during surgery - Teaching/training of CME procedure - Impact on operative time | - 1 = very good - 2 = good - 3 = satisfactory - 4 = sufficient - 5 = poor |
| Question 11) | Will you apply the “critical view/open book” concept *after* the surgical training course? | - Yes - Undecided - No, because: free-text response (optional) |
| Question 12) | What did you like most about the “critical view/open book” concept? | Free-text response |
| Question 13) | What did you dislike about the “critical view/open book” concept? | Free-text response |
| Question 14) | Do you have any additional comments or suggestions? | Free-text response |
